# Transcranial Magnetic Stimulation in the Treatment of Neurological Diseases

**DOI:** 10.3389/fneur.2022.793253

**Published:** 2022-05-20

**Authors:** Fahad A. Somaa, Tom A. de Graaf, Alexander T. Sack

**Affiliations:** ^1^Department of Occupational Therapy, Faculty of Medical Rehabilitation, King Abdulaziz University, Jeddah, Saudi Arabia; ^2^Section Brain Stimulation and Cognition, Department of Cognitive Neuroscience, Faculty of Psychology and Neuroscience, Maastricht University, Maastricht, Netherlands; ^3^Center of Integrative Neuroscience, Maastricht University, Maastricht, Netherlands; ^4^Department of Psychiatry and Neuropsychology, School for Mental Health and Neuroscience, Brain + Nerve Centre, Maastricht University Medical Centre+, Maastricht, Netherlands

**Keywords:** transcranial magnetic stimulation (TMS), Alzheimer, Parkinson, movement disorder, epilepsy, migraine, stroke

## Abstract

Transcranial Magnetic Stimulation (TMS) has widespread use in research and clinical application. For psychiatric applications, such as depression or OCD, repetitive TMS protocols (rTMS) are an established and globally applied treatment option. While promising, rTMS is not yet as common in treating neurological diseases, except for neurorehabilitation after (motor) stroke and neuropathic pain treatment. This may soon change. New clinical studies testing the potential of rTMS in various other neurological conditions appear at a rapid pace. This can prove challenging for both practitioners and clinical researchers. Although most of these neurological applications have not yet received the same level of scientific/empirical scrutiny as motor stroke and neuropathic pain, the results are encouraging, opening new doors for TMS in neurology. We here review the latest clinical evidence for rTMS in pioneering neurological applications including movement disorders, Alzheimer's disease/mild cognitive impairment, epilepsy, multiple sclerosis, and disorders of consciousness.

## Introduction

Transcranial magnetic stimulation (TMS) is a non-invasive, safe and painless procedure to activate or modulate cortical targets in the central nervous system (CNS) (1, 2). TMS is based on Faraday's law of electromagnetic induction, whereby an electrical current is discharged into a TMS coil, generating a perpendicular magnetic field that transcranially and thus noninvasively reaches the brain where it, due to its time-varying characterstics, generates an electric field and electrical currents in the targeted brain tissue (3, 4). If sufficiently strong, such induced electrical currents depolarize the neurons and result in TMS-induced action potentials (neural firing) measurable with electroencephalogram (EEG) (5) and/or with motor evoked potentials(MEPs) (2, 6), or also indirectly with functional magnetic resonance imaging (fMRI) (7).

The effects of rTMS on cortical excitability depend on the precise parameters selected in the so-called rTMS protocols (Table 1) as well as coil geometry (Table 2) (8). As a rule of thumb, low frequency [LF ≤ 1 hertz (Hz)] rTMS decreases cortical excitability and high frequency rTMS (HF ≥ 5–20 Hz) increases excitability (6). For example, when applied to the motor cortex, LF-TMS reduces MEP amplitudes and increases the duration of the cortical silent period, while HF-rTMS leads to opposite effects.

**Table 1:** Types of TMS pulses (2, 8).

**Types of pulse**	**Definition**
sTMS	Discharge of single pulses to a specific brain region separated by a time interval in the order of seconds.
Double pulse/Paired-pulse TMS	Two paired pulses with identical or different intensities, separated by an interval in the order of milliseconds
rTMS	Delivering any combination of more than two pulses with a time interval of ~ ≤ 2 s to generate different effects from those produced by an isolated pulse. Two categorical types: low-frequency rTMS (around 1 Hz) or high-frequency rTMS (around ≥5 Hz; typically 10 Hz)
TBS	A type of rTMS characterized by the application of 50 Hz bursts of 3 pulses applied every 200 ms. Two categorical types: (a) Continuous TBS (inhibitory): (conventionally) 40 s of TBS, meaning 600 pulses in total. (a) Intermittent TBS (excitatory): 2 s trains of TBS separated by 8 s of no stimulation, with 600 pulses in total.

*LTD, long-term depression; LTP, long-term potentiation; MEP, motor evoked potential; ms, milliseconds; rTMS, repetitive transcranial magnetic stimulation; sTMS, Single pulse TMS; TBS, theta burst stimulation*.

**Table 2:** Types of coils (9–12).

Circular coil	Non-focal, ring-shaped coil; stimulates a broader region of the brain.
Figure-8 coil	A pair of adjacent circular loops with current flowing in the opposite direction; focused electric stimulation below the point where the two rings intersect each other
Cloverleaf coil	Four coils of nearly circular windings; stimulates long fibers better than figure-8 coils
Slinky coil	Multiple circular or rectangular loop windings joined together at one edge and fanned out at other edge to form a half toroid; larger field magnitude and better focus near the coil center
Three-dimensional (3-D) differential coil	Small figure-8 coil with a third loop present perpendicular to its center and surrounded by two additional loops to limit the area of stimulation; more focal stimulation than figure-8 and slinky coils
Double cone coil	Two large adjacent circular windings fixed at an angle to each other; deeper stimulus penetration than figure-8 coil but a less focal electric field
Hesed (H) coil	More complex winding patterns and larger dimensions than conventional TMS coils, the H coils can stimulate deeper brain regions more effectively but at the expense of decreased focality.
Triple halo coil (THC)	The THC can deliver significantly greater E-Field intensities to deep brain regions than conventional TMS coils while avoiding critical regions such as optical nerves, eyes, retina and brain stem. The design is aimed to maximize the depth of stimulation, without concern for focality; the deep regions are stimulated with lesser intensity.
Other coil designs	The C-core coil, circular crown coil, the large halo coil, and MRI gradient coil designs with larger dimensions than conventional and H coils have also been under investigation for deeper TMS with the expectation of slower electric field decay at the expense of reduced focality

TMS helps to study the neural pathways in various CNS pathologies. Single pulse TMS (sTMS) evokes immediate sensory or motor responses and can therefore help assess the efficacy or speed of conduction of a particular neural pathway (1). Repetitive TMS (rTMS) modulates brain function in such a way that effects last beyond the period of stimulation. The magnetic and electrical fields generated by rTMS bring about many changes in the human brain that may confer therapeutic benefit (2). For instance, since rTMS can have lasting effects on cortical excitability through induced synaptic plasticity mechanisms, it is likely to help in the treatment of various psychiatric and CNS disorders where cortical excitability is one of the primary underlying pathologies (1, 2, 6, 8, 13).

The therapeutic potential and applications of rTMS have received much attention in recent years. Especially in the field of psychiatry, rTMS is now a widely recognized and applied treatment option for the therapy of major depressive disorder (14, 15) or obsessive compulsive disorder (16, 17) and shown to be clinically effective and reimbursed by health insurances (18, 19).

The clinical applications of TMS seem to be less successfully applied in neurology, which is somehow surprising and not necessarily straightforward, as TMS is deeply rooted as a diagnostic technique in neurology and clinical neurophysiology (20–23). It is therefore encouraging to see that in the recent update of the evidence-based guidelines by Lefaucheur (24) on the therapeutic use of rTMS, two neurological applications (neurorehabilitation after motor stroke and the treatment of neuropathic pain) received the highest level of evidence rating, namely “level A evidence” (definite efficacy). This rating was on par with the rating used for TMS application in depression treatment.

There are other currently applied rTMS treatments in neurology, for instance in the acute treatment of migraine and migraine prevention, non-motor stroke, other CNS pain syndromes, and H-coil deep TMS for poststroke aphasia (6, 25, 26). But beyond motor stroke and neuropathic pain, few rTMS protocols and applications have yet received the same level of scientific/emperical scrutiny. For some potential applications, there is no recommendation, or classification of efficacy, based on evidence from randomized controlled trials, systematic reviews and meta-analyses (27). However, it is important to realize that the lack of a formal recommendation, or classification of efficacy, does not necessarily mean that an rTMS application has no promise. Sometimes, the lack of such a recommendation is due to underpowered, or inconsistent, evidence from clinical studies. However, other times, the evidence that exists is in fact very promising, but not yet of sufficient size or scope that recommendations are warranted. It is therefore crucial to continue monitoring the state of evidence for these less investigated yet pioneering rTMS applications. Here, we focus on those “other” or “underinvestigated” neurological disorders, by reviewing the latest clinical evidence for the potential of rTMS in the treatment of movement disorders, Alzheimer's disease/mild cognitive impairment (MCI), epilepsy, multiple sclerosis (MS), and disorders of consciousness.

## Literature Search Strategy

General literature search, Google Scholar and MEDLINE search was carried out until December 25, 2020, by using the following search terms in various combinations: “transcranial magnetic stimulation,” “treatment,” “neurological diseases,” “Alzheimer's disease,” “Parkinson's disease,” “post stroke,” “multiple sclerosis,” “epilepsy,” “dystonia,” “Tourette syndrome,” “chronic tic disorders,” “Huntington's disease,” “choreas.” Only English language publications covering therapeutic benefits and challenges of TMS in neurological conditions of the CNS were considered. Literature covering pathophysiological and diagnostics aspects of TMS were excluded. Similarly studies looking at therapeutic benefits and challenges in neurobehavioral, psychiatric and chronic pain conditions were not included in the narrative review.

## TMS in Movement Disorders

rTMS has been shown to bring about some level of improvement in movement disorders, such as Parkinson's disease (PD), dystonia, Huntington's disease, and Tourette syndrome (Table 3) (8).

**Table 3:** TMS in Movement disorders.

	**rTMS protocol**	**Efficacy**
**Parkinson's disease**		
Gait and bradykinesia (28)	M1- and DLPFC-bilaterally 25 Hz at 100% rMT Sham-rTMS	Significant improvement in gait and reduction in bradykinesia of upper limb were found, lasting for at least 1 month after treatment ended.*
Motor performance (29)	M1-bilaterally 25 Hz at 100% rMT: Early PD M1-bilaterally 25 Hz at 100% rMT: Advanced PD M1-bilaterally 10 Hz at 100% rMT Mid-occipital 25 Hz at 100% rMT	Significant improvement in total motor functions (UPDRS), walking speed and key tapping were found.* The effect at 10 Hz was less significant than that at 25 Hz rTMS and was maintained for 1 month after the treatment.
Motor performance (30)	M1-bilaterally 25 Hz at 100% rMT	Significant improvements in total motor functions (UPDRS) and in serum dopamine level were found *Moreover, a significant correlation between serum dopamine level and motor functions was found before and after treatment.
Bradykinesia (31) Hamada et al. (31)	SMA-bilaterally 5 Hz at 110% aMT Sham-rTMS	Significant improvement in bradykinesia was found.* The effects of rTMS lasted for at least 2 weeks after the end of the treatment.
LID (32)	M1-L or R 1 Hz at 90% rMT Sham-rTMS	No significant differences were found. However, when compared to the baseline, a small but significant reduction in dyskinesia was found in favor of 1Hz-rTMS.*
Motor (33)	M1-L or R 1 Hz at 90% rMT Sham-rTMS	No significant differences were found
Motor; 20 studies, 470 patients (34)	Different rTMS protocols	Pooled SMD 0.46 (95% CI, 0.29-0.64), overall medium but significant effect size in reducing motor symptoms favoring active rTMS over sham (*P* < 0.001) Significant effect sizes of HF- rTMS targeting the m1 (SMD, 0.77; 95% CI, 0.46-1.08; *P* < 0.001) and LF-rTMS applied over other frontal regions (SMD, 0.50; 95% CI, 0.13-0.87; *P* = 0.008)
Motor (35) 11 randomized sham controlled trials; 246 patients	Different rTMS protocols	M1 targeting significantly improved UPDRS III scores at the short-term follow-up (Cohen's d of 0.27, UPDRS III score improvement of 3.8 points) but not during long-term follow-up No significant improvement in the UPDRS II
Motor (36)	Single session dual-site rTMS (1 Hz) directed to PMd and M1 (“ADS-rTMS”)	No significant improvement in Parkinsonian motor symptoms: videography of MDS-UPDRS-III, finger tapping, spectral tremor power. Variation of the premotor stimulation site did not induce beneficial effects
LID (37)	rTMS (5 Hz) bilaterally over the motor hand and leg areas of the cortex; 20 trains; 100 pulses in each train with 20-s inter-train interval	Significant improvement in LID after rTMS (*P* < 0.001), but no improvement in sham (*P* = 0.585). rTMS caused significant improvement of painful dyskinesia (*P* = 0.046)
PD with dysphagia (38)	rTMS (2,000 pulses; 20 Hz; 90% rMT; 10 trains of 10 s with 25 s between each train)	Significant improvement on all dysphagia rating scales; Significant and long-lasting (3 months) effect of time on all subitems of the A-DHI (functional, *P* = 0.0001; physical, *P* = 0.0001; emotional, *P* = 0.02) in r-TMS but not in the sham group; Significant improvement in H1-H2 (*P* = 0.03) and PTT (*P* = 0.01) during solid swallows in rTMS but not the sham group
Freezing gait (39)	HF-rTMS over SMA	Significant improvement in freezing of gait biomarker (*p* = 0.0071) and PD biomarker (*p* = 0.0378) after rTMS
**Focal hand/arm dystonia**		
Primary focal dystonia (40)	1 Hz rTMS at 90% RMT to dPMC	No effects in global clinical score and handwriting performance
Writer's cramp (41)	0.2 Hz rTMS at 85% RMT to M1, PMC, SMA	Improved writing rating and pen pressure after PMC stimulation; Prolongation of the CSP after PMC stimulation
Handwriting performance (42)	1 Hz rTMS at 90% RMT to PMC	Improvement of handwriting performance that lasted for 10 days after treatment. These results were not observed after single sessions; Prolongation of the CSP
Writer's cramp (43)	1 Hz rTMS at 90% AMT to S1	Both subjective and objective (as measured by 20 min writing task) were detected 2 weeks after treatment. BFMDS did not change significantly; Increased task-related BOLD signal in superior parietal lobule in fMRI
Focal hand dystonia (44)	cTBS 3-pulse 50 Hz burst every 200 ms at 80% AMT for 40 s to PMC	All subjects (including those in the sham arm) reported a subjective improvement, but no significant changes were observed on two different writing tasks; Improved intracortical inhibition in M1
Focal hand dystonia (45)	1 Hz rTMS at 80% RMT to dPMC (2 cm anterior and 1 medial to FDI hotspot)	No additional benefit from sensorimotor retraining; Analyses across the group revealed significant improvement in self-rated changes with large effect size indicating clinical meaningfulness
Focal hand dystonia (46)	1 Hz rTMS at 80% RMT to dPMC (2 cm anterior and 1 medial to FDI hotspot)	No additional benefit from sensorimotor retraining; Analyses across the group revealed significant improvement in self-rated changes with large effect size indicating clinical meaningfulness
**Cervico-facial dystonia**		
Benign essential blepharospasm (BEB) (47)	15 min stimulation at 0.2 Hz with an intensity of 100% RMT. Three different stimulation conditions: using a circular coil, a Hesed coil and sham to ACC in the point of maximal MEP for the orbicularis oculi muscle (about 3.5 cm medial and 5.5 cm anterior to M1)	Significant improvement of all clinical outcomes (patient-based and clinician based) at the end and 1 h after the active stimulations. Similar results were obtained regardless of the type of coil
Cervical dystonia (48)	Two trains of cTBS were applied over the left and the right lateral cerebellum with a pause of 2 min between the two trains. Three pulse bursts at 50 Hz repeated every 200 ms for 40 s (600 pulses) were delivered over the lateral cerebellum at 80% AMT of the ipsilateral M1	Significant reduction of the TWSTRS for the real but not sham cTBS at the end of the stimulation period, but not at later follow-up of 2 and 4 weeks. A nonsignificant trend was observed for the BFMDS for the real but not sham cTBS In the cTBS group 2 weeks of cerebellar stimulation modified the CBI circuits over contralateral M1 at ISI = 10 ms in which CBI was reduced. Normalization of the excessive baseline facilitation as measured with the PAS protocol
Cervical dystonia (49)	0.2 Hz at 85% of RMT for 15 min (for a total of 180 pulses) to Left ACC, M1, dPMC, SMA and sham dPMC (interventions were guided by a neuronavigation system)	All sites except ACC showed non-significant improvement in TWSTRS scores with the greatest improvement seen over dPMC and M1
Cervical dystonia (50)	1 Hz rTMS at 90% RMT to Left M1 and S1 (2 cm posterior and 1 lateral to M1)	S1 and M1 rTMS had no influence on symptom severity;
**Huntington's disease**		
Motor symptoms (51)	900 pulses, HF-rTMS 18 trains of 50 stimuli at 5 Hz frequency separated by 40 s of pause, delivered at 110% rMT LF-rTMS: 900 pulses, 1 Hz set at 90% of the RMT Sham: coil was angled such that there was no current to the brain	Sham rTMS did not modify AIMs; 5Hz rTMS: No beneficial effect, except slight AIMs increase immediately after; rTMS of 1 Hz: significant reduction of AIMs in all patients; effect was still observable 30 min, but not after 45 and 60 min. one patient presented a transient bradykinesia worsening immediately after
Motor symptoms in sever Huntington's chorea (52)	Seven consecutive sessions of bilateral LF-rTMS to SMA	Not even a transient reduction in the intensity of choreiform movement
Motor symptoms and Mood (53)	M1-rTMS at different frequencies	10 Hz rTMS shortened cRT (left hand) and prolonged sRT (right hand) 1 Hz rTMS: sustained improvement in mood (unexpected study finding)
**Tourette syndrome (TS)**		
Tourette Syndrome (54)	rTMS 1,200 pulses in 1 session per day for 2 days, 1Hz, 80% AMT 2 week interval between sites; Left motor cortex Left premotor cortex Sham	No significant clinical improvement in: MOVES, HDS-D
Tourette Syndrome (55)	rTMS 1,800 pulses in 1 session per day for 2 days, 1Hz, 80% AMT 4 week interval between sites to Left + right premotor cortex Left premotor cortex + right premotor cortex sham Right + left premotor cortex sham	No significant clinical improvement in: YGTSS, MOVES, MRVS
Tourette Syndrome and OCD (56)	rTMS 1,200 pulses divided in four sessions per day over 10 days, 1 Hz, 100% rMT to bilateral SMA	Significant clinical improvement in: YGTSS, YBOCS, HDRS-24, HARS-14, CGI, SCL-90 BDI SAD, SASS
Severe Tourette Syndrome (57)	rTMS Phase 1: 1,800 pulses in 1 session per day over 15 days, 1 Hz, 110% rMT Phase 2: 1,800 pulses in 1 session per day over 30 days, 1Hz, 110% RMT to bilateral SMA	Phase 1: No significant clinical improvement in: YGTSS, YBOCS, PUTS, ASRS Phase 2: Significant clinical improvement in YGTSS

*Significance level at ≤ 0.05.

*ACC, anterior cingulate cortex; A-DHI, Arabic–Dysphagia Handicap Index; AIMs, Abnormal Involuntary Movement Scale; aMT, active Motor Threshold; ASRS, Adult ADHD Self Report Scale; BDI, Beck Depression Inventory; BFMDS, Burke-Fahn-Marsden Dystonia Rating Scale; CBI, cerebellar inhibition of motor cortex; CGI, Clinical Global Impression; cM1, contralesional M1; cRT, choice reaction times, CSP, cortical silent period; cTBS, continuous Theta Burst Stimulation; dPMC, dorsal premotor cortex; DLPFC, dorso lateral prefrontal cortex; H1-H2, maximal hyoid elevation; HARS-14, Hamilton Anxiety Rating Scale-14; HDRS-24, Hamilton Depression Rating Scale-24 item; HDS-D, Hospital Anxiety and Depression Scale; Hz, Hertz; iM1, ipsilesional M1; ISIs, interstimulus intervals; iTBS, intermittent Theta Burst Stimulation; L, left; LID, Levodopa induced dyskinesia; M1, primary motor cortex; MEP, motor evoked potential; MOVES, Motor tic, Obsessions and compulsions, Vocal tic Evaluation Survey; MT, motor threshold; MRVS, Modified Rush Video-Based Tic Scale; NHPT, Nine Hole Peg Test; OCD, obsessive-compulsive disorder; PTT, pharyngeal transit time; PUTS, Premonitory Urge to Tics Scale; R, right; RCT, randomized clinical trial; rMT, resting Motor Threshold; rTMS, repetitive transcranial magnetic stimulation; SAD, Seasonal Affective Disorder; SASS, Social Adaptation Self-evaluation Scale; SMA, supplementary motor area; SCL-90 BDI, sRT, simple reaction time; TBS, Theta Burst Stimulation; TWSTRS, Toronto Western Spasmodic Torticollis Rating Scale; UPDRS, Unified Parkinson's disease Rating Scale; Y-BOCS, Yale-Brown Obsessive Compulsive Scale; YGTSS, Yale Global Tic Severity Scale*.

### Parkinson's Disease

It has been suggested by experimental research that changes in neurotransmitter release, transsynaptic efficiency, signaling pathways and gene transcription are induced by rTMS (58–62). Additionally, current research suggests that repetitive transcranial magnetic stimulation (rTMS) stimulates neurogenesis, neuronal survival, and the release of neuroprotective chemicals in Parkinson's disease patients (58, 63–65). One possible mechanism of action may relate to high-frequency rTMS-enhanced activity in the caudate nucleus as well as a relief of dopamine deficiency in nigrostriatal-thalamo-cortical circuitry (66–68). For instance, rTMS over M1 seems to affect dopamine release in nigrostriatal regions (24, 69).

Literature shows that rTMS could potentially be used as an important adjunctive treatment for PD (Table 3) (8, 34, 35, 70). Bradykinesia and tremor are two of the most debilitating motor symptoms in PD and thought to be related to abnormal oscillations in the subthalamic nucleus (STN) (71). Literature suggests that rTMS, especially the bilateral delivery over motor cortical regions, helps in improving motor symptoms (8, 13, 24, 27, 70). In these patients, favorable targets for high-frequency repetitive transcranial magnetic stimulation (HF-rTMS) include primary motor cortex (M1), less focal motor cortex (MC) stimulation such as to leg or bilateral hand MC, and dorsolateral prefrontal cortex (DLPFC), while supplementary motor area (SMA) was found to be the most favorable low-frequency repetitive transcranial magnetic stimulation (LF-rTMS target) (8, 13, 70). rTMS to these targets has also been found effective for levodopa-induced dyskinesia (LID) (37). However, Lefaucheur (24) felt that these benefits were sometimes the results of a single session, and the prolonged clinical benefit needs to be investigated. Additionally, literature reporting the beneficial effect of HF-rTMS of the left DLPFC in treating non-motor depressive symptoms in PD has been covered in many review articles (8, 13, 70). However, a randomized trial failed to show any significant benefit in mood upliftment (24, 72).

In their evidence based guidelines on the therapeutic use of rTMS, Lefaucheur (24) suggest that of the various targets studied, M1 stimulation may be recommended for treating motor symptoms in PD with repeated HF-rTMS. A large double-cone coil applied to M1 leg area may help improve freezing-of-gait. However, specific recommendations for the use of rTMS in PD could not be made without further research.

Repetitive transcranial magnetic stimulation (rTMS) has been suggested as a potential treatment for cognitive impairment in Parkinson's disease (PD), with effects that appear to be additive to dopaminergic medicines (73). While it is difficult to pinpoint the exact role of pathological neural oscillations in certain aspects of motor and cognitive function, current research clearly suggests that these pathological oscillations interact and contribute to the motor and cognitive deficits seen in Parkinson's disease (74). Another study found that repetitive transcranial magnetic stimulation (rTMS) over (motor region) M1 is beneficial for motor function and may have a slight favorable effect on cognition (73). The efficacy of TMS on depression and cognition in Parkinson's disease has yielded promising preliminary results. Although it is unknown if these effects are transient, what the underlying processes are, and if such neuromodulation might transfer to real-world settings, a small study found that TMS can improve working memory in PD patients (75).

### Dystonia

The exact mechanisms of action of rTMS in alleviating dystonia remain unknown (76–80). Although motor cortex hyperexcitability appears to be the cause of aberrant co-contraction and overflow to adjacent muscles, several studies have shown that plasticity processes and integrated sensorimotor processing are also likely to be involved (76, 78, 81, 82).

Cortical hyperexcitability in dystonia is thought to be caused by two abnormalities in the sensorimotor system (83). The inhibitory systems are less excited and there is an increase in the plasticity of neural connections. Hence, rTMS may be a useful therapeutic tool for dystonia if it can increase intracortical inhibition and reduce excessive cortical plasticity. LF-rTMS and cTBS protocols (continuous theta burst stimulation; a patterned inhibitory rTMS protocol thought to have analogous effects to LF-rTMS) have been investigated in dystonia by targeting M1, PMC, SMA, primary somatosensory cortex, and cerebellum. Different protocols were used for writer's cramp and craniocervical dystonia (Table 3) (84). A literature review by Erro et al. (84) found mixed evidence of benefit. While some studies reported short-lasting objective or subjective improvement in dystonia, others did not (Table 3).

### Huntington's Disease

The dopaminergic system, particularly in the frontal brain, can be affected by rTMS. TMS can cause an increase in the flow of dopamine to numerous parts of the brain, including the nucleus accumbens and the dorsal striatum, due to the connection between dopaminergic pathways in the cortex and those sub cortical structures (85–88). These dopaminergic pathways are the likely mediators in the beneficial effects of rTMS in Huntington disease patients (85, 87, 88).

There are currently only very limited, very small, studies (<10 patients in a study) (Table 3) reporting inconclusive evidence of the beneficial effect of TMS in ameliorating motor symptoms in Huntington's Disease (51–53). SMA is believed to play a key role in maintaining the executive aspects of motor control in Huntington's Disease (89). A small study did report a benefit in uplifting mood (non-motor symptom) (53).

### Tourette Syndrome

Very little is known about mechanisms of action of rTMS in Tourette syndrome (90, 91). It was suggested that low frequency rTMS may help with tics and obsessive behaviors by resetting a hyperactive motor cortex (90, 92). But there are currently a limited number of rTMS studies in adult Tourette syndrome, overall showing mixed results (Table 3). Some LF-rTMS (1 Hz) and HF-rTMS (15 Hz) studies targeting motor and premotor cortical sites demonstrated no success or a limited benefit in severe Tourette syndrome (54, 55, 93). On the other hand, several open-label studies targeting SMA with LF-rTMS (1 Hz) demonstrated a decrease in the frequency and intensity of tics (56, 94–97).

### Essential Tremor

rTMS can regulate brain functions through plasticity effects and it has been targeted to the tremor network to achieve therapeutic effects (93, 98–101). One rTMS protocol that has been tested in clinical trials is LF-rTMS of the cerebellum (99, 102, 103). However, this protocol did not show any improvement in tremor variables in essential tremor (103) or in resting tremor in PD (104). Other researchers tried stimulating the left M1 or premotor cortical targets but did not find any appreciable benefit in tremor reduction (105, 106).

However, a double-blind sham controlled study (*N* = 10; five essential tremor, five sham) investigating LF-rTMS of the pre-SMA found significant reduction in tremors after 15 daily sessions. Though tremor reductions were also seen in the sham group (26% in essential tremor and 19% of patients in sham), sustained effects at 4 and 8 week follow-up were only seen in the essential tremor group (107).

### Gaps and Challenges

Of the various movement disorders discussed, rTMS may currently be considered an emerging strategy in ameliorating certain motor symptoms in Parkinson's disease, with moreover an effect in uplifting mood. There remains a need to increase the effectiveness of rTMS in Parkinson's disease by finding optimal stimulation strategies. When it comes to the other movement disorders covered here, the few studies showing therapeutic benefit of rTMS in dystonia seem too small to yield conclusive evidence. Though SMA has shown some promise as an effective target in Huntington's disease, also there, rTMS trials have been small and results inconclusive. The rTMS trials in Tourette syndrome show a lack of significant effects, raising doubt about the possible efficacy of rTMS. In tremor, though LF-rTMS to cerebellum and pre-SMA has shown some benefit in essential tremor, overall, the available data from small samples remains inconclusive. Larger, well- designed trials assessing rTMS efficacy in treating each of these disorders are required. Also, there is a need to reduce variability in the TMS protocols evaluated for any particular movement disorder. Another issue is that the same TMS protocol may give different results in different individuals (inter-individual variability) and also in the same individual at different times (intra-individual variability). In a recent article, our group has described the possible determinants causing these intra- and inter-subject variability, hindering its reliability, and efficacy. Among differences in general TMS reactivity due to differnces in, e.g., scalp-cortex distances or cortical excitability, recent findings suggest a systematic state-dependence of rTMS in which the cognitive but also spontaneous oscillatory brain state can modulate the size and direction of rTMS effects in the brain (108). Hopefully, the overview of currently available evidence provided here can help inform further clinical work.

## TMS in Alzheimer's Disease

Protocols of rTMS are based on persistently enhancing cortical excitability by repetitive high-frequency stimulation (109, 110). Long-term potentiation (LTP)-like changes in synaptic strength, which are commonly assumed to be a major cellular mechanism of learning and memory, are thought to be involved in such facilitation (109–111). The expression of plasticity-related neurotrophins like brain-derived neurotrophic factor (BDNF) which diminishes in the hippocampus of Alzheimer's disease patients, is regulated by neuronal activity and LTP (109, 112). Hence rTMS can considerably increase BDNF levels. By the correction or blunting of impaired LTP-like plasticity and associated signaling defects seen in AD, rTMS may provide clinical benefit (109, 113, 114). rTMS has also shown to be an inhibitory neuron function modifier as the studies show that GABAergic synaptic strength on principal neurons is reduced by 10 Hz stimulation, confirming a concept in which GABAergic synapses influence overall inhibitory/excitatory balance (109, 115, 116).

Two recent meta-analyses showed that rTMS may be an effective therapy to improve cognitive ability in patients with mild to moderate AD including MCI. In the first meta-analysis (15 RCTs; *N* = 240), rTMS was found to be an effective therapy to improve cognitive impairment in AD (117). rTMS significantly improved cognition in AD compared to sham (*P* = 0.0006). A subgroup analysis suggested that rTMS on multiple sites and multiple sessions (>10) provided more significant cognitive enhancement than rTMS on single site for ≤ 10 sessions. 20 Hz was more effective than 10 or 1 Hz frequencies. Concurrent cognitive training and/or patients with higher education seemed to confer higher benefit than single therapy, or in patients with lower education or severe dementia (117).

The other meta-analysis (12 studies including 8 RCTs; *N* = 231) also suggested that rTMS significantly improved cognition in AD compared to sham (*P* < 0.0001) (118). The sub-analysis moreover showed that multiple targets had better effects than single (0.86 vs. 0.13) and ≥5 sessions had better effect than ≤ 3 sessions (2.77 vs. 0.29). However, this meta-analysis did not find any benefit of concurrent cognitive training (118).

Thus, the meta-analyses showed that rTMS to multiple sites [Broca, right/left DLPFC, Wernicke, right/ left parietal somatosensory association cortex (pSAC), inferior frontal gyrus] and long-term treatment yields better cognitive performance than single site or short duration rTMS (109, 117, 118). However, while encouraging, this cannot be considered as a conclusive evidence as the studies included in both meta-analyses (Table 4) had small sample size, and some were not sham-controlled.

**Table 4:** TMS in Alzheimer's disease.

**Clinical feature**	**TMS Protocol**	**Efficacy**
Mild, moderate and severe AD (119)	One session of 20 Hz rTMS during cognitive stimulation to unilateral dlPFC and sham region.	Improved action naming accuracy during stimulation to either the right or left dlPFC
Moderate AD (120)	Two courses: 4 week stimulation Or 2 week placebo + 2 weeks stimulation. 20 Hz rTMS, for 25 min/d, 5 d/week to dlPFC (hemisphere not specified).	4 week rTMS: improved on SCBADA after the first 2 weeks. Placebo + 2 weeks rTMS: improved on SCBADA after the 2 weeks of stimulation. Effects lasted for 8 weeks in both groups
MCI (60)	rTMS vs. sham. 10 Hz for 5 s, 25 s intertrain interval 20 min/d for 5 d/week for 2 weeks to left dlPFC	rTMS: Improved RBMT scores lasting up for 30 d. Improved TMT-B 30 d after treatment. Sham: Improved logical memory (lasted 30 d), letter-number sequencing and TMT-B. Improved verbal fluency 30 d after treatment.
Mild or moderate AD (121)	rTMS-COG. Intensive + maintenance (4.5 months). 10 Hz for 2 s, 20 trains to Broca, right/left dlPFC, Wernicke, right/left pSAC	Significantly improved ADAS-cog scores after 6 weeks and 4.5 months.
Mild, moderate and severe AD (122)	rTMS vs. sham; 20 Hz: 5s, 20 trains OR 1 Hz: 2 trains of 1,000, 30 s intertrain interval. 5 d to bilateral dlPFC	20 vs. 1 Hz or sham: Improvement in all tests up to 3 months in mild to moderate AD 1 Hz vs. sham: Improved IADL in mild to moderate AD There was no improvement in severe AD.
Mild to moderate AD (123)	rTMS vs. sham; rTMSCOG. Intensive + maintenance (4.5 months). 10 Hz, 20 trains, for 2 s Broca, right/left dlPFC, Wernicke, right/ left pSAC	ADAS-cog and CGIC scores improved at the end of intensive phase. Effects lasted up for 4.5 months.
Mild to moderate AD (124)	DB rTMS vs. sham followed by OL maintenance; 20 Hz (40 pulses per burst) with 5-second intertrain intervals during cognitive task. 2,000 pulses to left and right DLPFC per session	DB: statistically significant changes on ADAS-cog or RMBC scores. Treated patients scored higher on MoCA in 2 and 3 weeks OL: All decline rates were better than the expected except for ADAS-cog scores for 2 patients.
Mild to moderate AD (125)	rTMS-COG. Intensive (6 weeks). 10 Hz, 20 trains for 2 s to Broca, right/left dlPFC, Wernicke, right/ left pSAC	Improved ADAS-cog and MMSE scores
Mild to moderate AD (126)	rTMS vs. sham; rTMSCOG. Intensive (6 weeks). 10 Hz, 20 trains for 2 s to Broca, right/left dlPFC, Wernicke, right/ left pSAC	Mild AD: Improved ADAS-cog sustained for 6 weeks, but not different from sham group. Improved MMSE 6 weeks after end of treatment. Sham: Improved GDS scores
Mild AD and moderate to severe AD (127)	rTMS-COG. Intensive + maintenance (4.5 months). 10 Hz, 20 trains, for 2 s to Broca, right/left dlPFC, Wernicke, right/left pSAC	Improved ADAS-cog, locomotor, apathy and dependence scores which returned to baseline 6 months after treatment.
Mild to moderate AD (128)	rTMS vs. sham; 20 Hz, 20 s intermediate/train. 1 session/day, 5 d/week for 6 weeks to parietal P3/P4 and posterior temporal T5/T6	Improved ADAS-cog, MMSE, MoCA and WHOUCLAAVLT. 6 weeks FU: Further improvement in ADAScog and WHO-UCLA AVLT. Sham: Improved on ADAScog compared to pretreatment.
Mild AD (129)	rTMS vs. sham (crossover); 2 weeks of 20 Hz stimulation (40 trains, for 2 s, 1,600 pulses/d) to Precuneus	Improved Delayed Recall of RAVLT
MCI vs. healthy controls (130)	iTBS/1Hz vs. sham. Control: 1 Hz and iTBS to unilateral dlPFC; MCI: 1 Hz bilateral dlPFC for MCI (3 weeks interval). iTBS: 20 trains, three 50 Hz pulses repeated at 5 Hz for 2 s. 1 Hz: 600 pulses	1 Hz to right dlPFC: Recognition memory improved in controls and MCI Healthy controls: iTBS over right dlPFC impaired nonverbal recognition memory. iTBS over left dlPFC had no effect
MCI and mild AD (131)	Two sessions of 10 Hz, 45 trains of 4.9, 25 s interval, 2,250 pulses/session to right inferior frontal gyrus and right superior temporal gyrus (rTMS), and vertex (sham). One-day interval between sessions	Inferior frontal gyrus: significant improvement in the TMT A and B. Right inferior frontal gyrus: No significant difference in the Stroop test or CVSET

*ADAS-cog, Alzheimer's Disease Assessment Scale–Cognitive Subscale; CGIC, Clinical Global Impression of Change; CVSET, complex visual scene encoding task; DB, double blind; DLPFC, dorso lateral prefrontal cortex; GDS, Geriatric Depression Scale; Hz, Hertz; IADL, Instrumental Activity of Daily Living; iTBS, intermittent Theta Burst Stimulation; MCI, mild cognitive impairment; MMSE, Mini–Mental State Examination; MoCA, Montreal Cognitive Assessment; OL, open label; pSAC, parietal somatosensory association cortex; RBMT, Rivermead Behavioral Memory Test; rTMS, repetitive transcranial magnetic stimulation; SCBADA, auditory sentence comprehension subtest from the Battery for Analysis of Aphasic Deficits; TMT, Trail Making TestB; WHO-UCLA AVLT, World Health Organization-University of California-Los Angeles Auditory Verbal Learning Test*.

### Gaps and Challenges

There is no recommendation yet for therapeutic use of TMS in AD and MCI (109). There are limited studies showing long-term efficacy. The clinical trials reporting positive effects on cognitive outcome measures in AD are very small and there are no clear neurobiological mechanisms to explain the benefit of rTMS in AD (109). In their evidence based guidelines, Lefaucheur (24) note that multisite rTMS with concurrent cognitive training in AD may possibly improve cognition, memory, apathy, and language in mild and early stage AD (including MCI). However, they do not recommend its clinical use until long-term observational studies show that multisite rTMS with cognitive training is more beneficial than single-site focused rTMS with cognitive training. They also stress the need for neurophysiological and imaging studies to improve the understanding of the neural mechanisms of action. Additionally, a TMS strategy that may show positive effects in young adults may have detrimental effects in older adults or in patients with brain affected by AD pathology, so one should proceed with caution.

## TMS in Multiple Sclerosis

MS is usually treated with disease-modifying therapies. However, despite treatment, patients develop relapsing/remitting MS (RRMS) and secondary progressing MS (SPMS). Since TMS has no known interaction with MS drugs, it can be used as an adjunctive treatment for management of motor and sensory symptoms of MS (8). It is believed that some of the MS symptoms are related to neuronal transmissionin the brain (6). LF-rTMS of a single neuron can cause prolonged inhibition of neuronal transmission while HF-rTMS can improve neuronal transmission (132) Thus trains of rTMS pulses modify activity in the targeted region of brain lasting for minutes or even hours (132). Thus, TMS may alleviate debilitating MS symptoms such as fatigue, spasticity, and gait abnormalities and manual dexterity, which affect quality of life (QoL), especially in patients with RRMS and SPMS (Table 5) (2, 62).

**Table 5:** TMS in multiple sclerosis.

**Clinical feature**	**TMS Protocol**	**Efficacy**
Spasticity in RRMS (133)	LF-rTMS and HF-rTMS	A single session of 1 and 5 Hz rTMS over the leg primary motor cortex increased and decreased H/M amplitude ratio of the soleus H reflex, respectively; 5Hz rTMS also increased corticospinal excitability. When rTMS applications were repeated during a 2-week period, there was a significant improvement of lower limb spasticity; 5 Hz rTMS resulted in long-lasting clinical improvement was (at least 7 days after the end of treatment)
Lower limb spasticity (134)	iTBS	Compared to sham, iTBS showed a significant reduction of H/M amplitude ratio and MAS scores 1 week after the stimulation that persisted up to 2 weeks after the end of stimulation protocol.
Lower limb spasticity (135)	iTBS	iTBS group showed significantly better improvement in spasticity than sham iTBS group (*p* = 0.026). “iTBS had a significant effect on the balance of the connectivity degree between the stimulated and the homologous primary motor cortex (*p* = 0.005).” Changes in inter-hemispheric balance were significantly associated with improvement of spasticity (rho = 0.56, *p* = 0.015).
Motor performance (primarily spasticity and fatigue) (136)	iTBS + ET	iTBS plus ET reduced MAS, MSSS-88, FSS scores; physical composite scores were increased in the Barthel index and MSQoL-54
LUT dysfunction (133)	5-Hz rTMS motor cortex stimulation, five consecutive days	Ameliorates the voiding phase (detrusor contraction and/or urethral sphincter relaxation) of the micturition cycle
Motor performance (manual dexterity) in MS patients with cerebellar impairment (137)	5-Hz rTMS	rTMS improved hand dexterity in patients with cerebellar symptoms but not in healthy subjects
Dexterity in RRMS and SPMS (138)	HF-rTMS to motor cortex (two sessions)	Significant improvement in the time required to finish the pegboard task (*P* = 0.002) and cerebellar FSS (*P* = 0.000) seen after the second session and persisted 1 month later. RRMS patients showed more improvement than the SPMS patients.
Cognitive performance (working memory) Hulst et al. (139)	HF-rTMS	rTMS may have a role in cognitive rehabilitation in MS; rTMS significantly improved N-back task accuracy (N2 and N3) compared to sham (*p* = 0.029 and *p* = 0.015, respectively; At baseline, MS patients had higher task-related frontal activation (left DLPFC, N2 > N0) compared to healthy subjects, which disappeared after rTMS
Gait (140) case report; Caucasian male 51 years with chronic RRMS and residual disabling attention and gait symptoms	HF-rTMS (6 Hz) to left PFC at 90% MT using figure of 8 coil	Gait measured using GAITRite gait analysis system After three consecutive daily sessions there was significant improvement in ambulation time, gait velocity, and cadence

*AMT, active motor threshold; DLPFC, dorsolateral prefrontal cortex; EDSS, Expanded Disability Status Scale; ET, exercise therapy; fMRI, functional magnetic resonance imaging; FSS, Fatigue Severity Scale; iTBS, intermittent theta burst stimulation; LUT, lower urinary tract; MAS, Modified Ashworth scale; MS, multiple sclerosis; MSQOL-54, 54 item Multiple Sclerosis Quality of Life; MSSS-88, Multiple Sclerosis Spasticity Scale 88, 88-item Multiple Sclerosis Spasticity Scale; PFC, prefrontal cortex; RMT, resting motor threshold; RRMS, relapsing-remitting multiple sclerosis; rTMS, repetitive transcranial magnetic stimulation; SPMS, secondary progressive multiple sclerosis*.

Agüera et al. (141) reported a case of RRMS (33 years, female) not responding to medications prescribed over 9 years and rapidly progressing disease. The patient benefited from rTMS which was prescribed as a compassionate treatment as no other treatment was producing any benefit. Post rTMS, there was improvement in her neuropsychological functions and blood tests showed a reduction in oxidative stress after 4 months of treatment (141).

There is mixed evidence of benefit in fatigue. In 34 patients with secondary progressive MS, HF-rTMS (20 Hz) and intermittent TBS (iTBS), a patterned excitatory protocol with after-effects analogous to HF-rTMS was used for spasticity management. HF-rTMS and iTBS significantly showed significant reduction in spasticity on the Modified Ashworth Scale compared to sham stimulation (142). Intermittent theta burst stimulation (iTBS) had longer-lasting effect on the Subjective Evaluating Spasticity Scale (SESS) and when given after HF-rTMS resulted in reduction in pain and fatigue. However, a systematic review and meta-analysis comparing transcranial direct current stimulation (tDCS), TMS, and transcranial random noise stimulation (tRNS) did not find TMS to be beneficial in fatigue (143). The analysis included 207 patients from 14 studies (11 for tDCS, 2 for TMS, and 1 for tRNS). The analysis reported that tDCS had significant short-term and long-term treatment effects compared to sham stimulation but TMS and tRNS were not found to be superior to sham stimulation (143). However, Gaede et al. (144) reported some benefit of H-coil HF-rTMS deep brain stimulation to motor cortex in 37 patients with MS related fatigue. There was significant sustained median Fatigue Severity Scale (FSS) decrease of 1.0 point (95%CI, 0.45, 1.65). However, some participants discontinued treatment due to minor side effects and the study size was too small to make any conclusive suggestion.

### Gaps and Challenges

There is no conclusive recommendation yet for therapeutic use of TMS in MS. In their evidence based guidelines, Lefaucheur (24) suggests that iTBS targeted to the leg motor cortex may be recommended to treat lower limb spasticity in MS. However, they do not recommend using iTBS to the hand motor cortex for improving manual dexterity. Nor do they recommend using H-coil HF-rTMS deep brain stimulation to motor cortex to improve fatigue. Since iTBS and H-coil HF-rTMS has shown some benefit in MS, large studies with set iTBS and H-coil HF-rTMS protocols in MS will be required to identify how TMS can be effectively, therapeutically and routinely used in MS.

## TMS in Epilepsy

Though antiepileptic drugs (AEDs) are the mainstay of epilepsy treatment, one third of patients on AEDs develop drug resistance. Of these, many patients are not suitable candidates for surgical ablation. This patient group, which is at increased risk of morbidity, may respond to LF-TMS (8). rTMS could reduce likelihood of seizures in this patient population probably due to its ability to cause prolonged inhibitory effect on synaptic potential and focal cortical excitability (6). TMS has also been used to study the effects of AEDs on the brain (145). In patients who are candidates for surgical ablation, TMS helps identify the brain areas which are more seizure prone. Alternatively, TMS helps identify areas of cortical excitability in various epilepsy syndromes (145).

A Cochrane review of seven pilot studies from different regions of the world showed that, in all studies, TMS was used in patients with drug-resistant epilepsy (146). However, the definition of drug resistance differed between studies and ranged from ≤ 1 complex partial/secondarily generalized seizure per month to ≥3 seizures per month. Additionally patient should have had ≤ 2 unchanging AEDs. All studies used figure 8 coil though the sham TMS methods varied (146).

A meta-analysis of 11 studies (*n* = 164) evaluating the efficacy of LF-rTMS in medically intractable epilepsy found a significant effect size in seizure frequency [effect size: 0.34, 95% confidence interval (CI) 0.10-0.57] (147). Seizure reduction was significantly higher in patients with neocortical epilepsy or cortical dysplasia than those with other epileptic disorders (effect size of 0.71 vs. 0.22) (147).

In their systematic review, Cooper et al. (132) included 12 studies in patients with drug-resistant epilepsy being treated with LF-rTMS. Meta-analysis of the five studies with individual participant data (IPD) (*n* = 34) showed that patients with temporal seizure focus had significantly more favorable response than patients with extratemporal epilepsy (50 vs. 14%, *p* = 0.045). Stimulation with a figure-8 coil resulted in significantly more favorable response than stimulation with other types of coils (47 vs. 0%, *p* = 0.01). Meta-analysis of seven studies without IPD (*n* = 212) showed that seizure reduction rates were significantly higher in patients with mean age ≤ 21 years than those older than 21 years (69 vs. 18%) and in patients treated with targeted stimulation vs. those treated without targeted stimulation (47 vs. 14–20%). The pooled rate of 50% seizure reduction with LF-rTMS was 30% (95% CI 12–57%) (132).

### Gaps and Challenges

There is no recommendation yet for therapeutic use of TMS in epilepsy. Though the Cochrane review found rTMS to be safe and effective in reducing epileptiform discharges, the review could not find clear evidence of the efficacy of rTMS in reducing seizure frequency (146). There is currently too much variability in the TMS techniques used in studies, in the outcomes reported and in the definition of drug-resistant epilepsy (147, 148).

## TMS in Disorders of Consciousness

Disorders of consciousness (DOC) mainly include minimally conscious state (MCS) and the “vegetative state,” clinically known as the unresponsive wakefulness syndrome (UWS). The clinical efficacy of rTMS has been studied in these patients using different HF-rTMS protocols, mainly targeting the left M1 (149–151) and the right and left DLPFC (152–154) or iTBS to left DLPFC (155). Cincotta et al. (149) (*N* = 11 patients with UWS) and Liu et al. (150) (N-7 patients with DOC and 11 healthy controls) tested 20 Hz rTMS of the M1 but found no evidence of therapeutic effect. He et al. (151) posted results of a randomized sham controlled study of six patients with DOC treated with 20 Hz rTMS of the M1 for five consecutive days. rTMS resulted in long-lasting behavioral and neurophysiological modifications in one patient with traumatic brain injury while five other patients showed localized brain reactivity at several electrodes, but no significant electroencephalography changes. This was a very small study with inconclusive evidence of efficacy of HF-rTMS pf M1 in DOC.

Similarly, targeting DLPFC did not provide any conclusive evidence of effect in DOC. Naro et al. (152) (*N* = 10 postanoxic UWS and 10 healthy controls) did not find any significant clinical change after a single session of 10 Hz rTMS to the right DLPFC. However, three patients showed short-lasting clinical improvement caused by a significant transient effect induced by rTMS (152). On the contrary, Xia et al. (153), found clinically significant benefit of 10-Hz rTMS to the left DLPFC for 20 consecutive sessions in 16 patients (5 MCS and 11 UWS). In another 2017 study, Xia et al. reported reduced EEG signal power in low-frequency and increased signal power in the high-frequency bands (154).

A study using iTBS (600 pulses per session at 80% of active motor threshold) to the left DLPFC for 5 consecutive days in eight patients with MCS or UWS reported some clinical benefit after rTMS but the benefit was statistically significant only after a week. This was a small study with no sham control.

### Gaps and Challenges

There is no recommendation yet for therapeutic use of TMS in DOC. Though some clinical benefit in consciousness level was seen using HF-rTMS of the left M1 or after HF-rTMS or iTBS of the left DLPFC, the small sample size of these studies limits the generalization of the results. Also, contrary results from other studies raise a level of doubt regarding efficacy in DOC. Further studies testing the efficacy of TMS protocols to left M1 or DLPFC are required to determine whether TMS has any efficacy in DOC.

## Safety of TMS in Neurological Conditions

TMS is a relatively safe procedure. A systematic review of 93 RCTs found the TMS group had 2.60 times higher (95% CI 1.75 3.86) odds of experiencing an adverse event (AE) than placebo (*p* < 0.00001). Headache and dizziness were the most common AEs. However, the overall pooled estimate of treatment discontinuation due to an AE was 2.5% (95% CI 1.9-3.2%) with TMS and 2.7% (95% CI 2.0-3.5%) with placebo (156).

A meta-analytic utility prediction study including 35 studies investigating treatment of focal epilepsy (*N* = 6,398; 28 AEDs and 7 rTMS studies; AEDs *n* = 4,919; rTMS *n* = 136 and placebo *n* = 1,343) found that adjunctive rTMS provided superior QoL as compared to AEDs (148). However, there was no difference in seizure reductions between AEDs and rTMS (*p* = 0.94). Reduction of seizure frequency from baseline to final treatment follow-up with AED, rTMS and placebo was 36.1 ± 15.2%, 36.2 ± 7.2% and 19.6 ± 8.5%, respectively. The superior QoL was due to fewer side effects, most of which were considered mild (148). There was no difference in adverse effects between rTMS and sham TMS groups. This suggests that adjustments in the treatment environment may have mitigated the TMS related adverse effects. On the other hand, adverse effects were the main reason for treatment discontinuations in the AEDs arms of the studies (148).

A Cochrane review of seven studies analyzing the effect of TMS in epilepsy found that adverse effects were uncommon; most reported adverse effects were headache, dizziness, and tinnitus and did not lead to a significant change in medications (146). A meta-analysis of 17 RCTs evaluating rTMS to left DLPFC (10 Hz) at 60-110% resting motor threshold (rMT) reported a significant incidence of headache in the treatment group.

TMS use in epilepsy may induce seizures in patients with a known neurological disorder and clinicians should be aware and alert about this complication (6, 157). However, the risk is very low, most incidences are transient and self-limiting and do not have any long-term sequelae (157).

## Clinical Effects of rtms in Cerebellar Ataxias

Cerebellar low frequency TMS works by lowering the inhibitory regulation of the cerebellar cortex over the dentate nucleus, hence potentiating some of the impaired functionality of dentate nucleus. Furthermore, a reduced inhibitory signal from Purkinje cells may boost the activation of the vestibular nuclei, resulting in improved balance in patients with cerebellar ataxias (158–162).

### Spinocerebellar Ataxia

Dysfunction of the cerebellum and its connected neural networks causes a neurodegenerative disorder known as spinocerebellar ataxia (SCA). In a randomized, double-blinded and sham-controlled study significant improvement in clinical and kinematic outcomes of postural control in standing were observed in patients who completed a 4-week rTMS intervention with 1-month follow up as compared to the patients receiving sham intervention (159).

### Hereditary Ataxias

The role of TMS in diagnosis, pathophysiology and treatment interventions of genetically confirmed hereditary ataxias was studied in a critical review (23). Hereditary ataxias are a heterogeneous group of neurodegenerative disorders affecting motor cortex and the corticospinal tract. Early involvement of the corticospinal tract and motor cortex circuitry was shown by the available data and the effectiveness of cerebellar repetitive TMS (rTMS) as treatment approach was observed (23).

### Truncal Ataxia

The efficacy of TMS over the cerebellum for inherited spinocerebellar degeneration was reported in a placebo-controlled trial (107). Patients treated with active TMS showed a significant reduction in truncal ataxia. The contraction of nuchal and shoulder muscles was evoked by active stimulation. Sham stimulation, on the other hand, generated the same noise as active stimulation, as well as some scalp sensation. The study's findings revealed that the disease type had an impact on TMS's effectiveness (163).

A study of Ihara et al. (164) compared pre and post severity of ataxia, cerebellar hemispheric blood flow (CHBF), ascorbate free radical (AFR), superoxide dismutase protein, superoxide scavenging activity, and 8-hydroxy-2′-deoxyguanosine (8-OHdG) in cerebrospinal fluid (CSF) during an 8-week course of repetitive transcranial magnetic stimulation (rTMS) in 20 individuals with spinocerebellar degenerations (SCD). After applying rTMS, AFR and ataxia severity decreased in SCD patients and CHBF increased.

### Neurodegenerative Ataxia

A review of Alberto et al. (165) concluded that non-invasive brain stimulation has made substantial advances in developing particular protocols of stimulation to regulate cerebellar excitability with the aim to restore the cerebellar physiological activity in ataxia patients. Literature showed that rTMS or tDCS may be useful tools for patients suffering from neurodegenerative ataxia.

## Placebo Effects

In rTMS, there are substantial placebo effects. This means that efficacy of rTMS (response rates, remission rates, etc.) should be evaluated not just in isolation, but in comparison to placebo groups. Therefore, clinical studies without good blinding and placebo control provide limited information on the extent to which clinical outcomes are attributable to direct neuromodulation effects or to indirect placebo effects. This is not only highly relevant in psychiatric applications, but also in neurology as, e.g., very evident in essential tremor. Most of the studies included in this review are placebo controlled. But it remains the case that some literature on neurological disorders lacks proper controls/blinding.

## Limitations and Strengths of the Review

This review is limited by its narrative structure. There is a high possibility of study selection bias. However, it is a comprehensive review of literature and its judicious interpretation that covers all aspects of TMS that a clinician would require for selecting the right patient population and TMS strategy for either investigating or treating a particular neurological disorder.

## Future Prospects and Summary

Several animal studies are being conducted and cerebellar stimulation is being explored to treat movement disorders (166). However, in general, TMS therapy for a particular neurological condition needs more directional exploration by standardizing study designs, end points, TMS frequency, target, coil, location of stimulus and other such variables.

This review shows that though TMS is not the first line treatment in the discussed neurological conditions, it has an important place in ameliorating symptoms and improving QoL of patients with debilitating disease not responding to drug therapy. With right patient, target and strategy selection, as summarized in Table 6, the required efficacy may be seen. However, it is too early to unambiguously recommend TMS as a therapeutic clinical option in many of these neurological conditions. To reach that stage, more clinical studies are necessary. By providing the current overview, hopefully we could contribute to informing those studies.

**Table 6:** The patient population and TMS strategy that may provide benefit and needs to investigated further in larger trials.

**Neurological condition**	**Patient group likely to get maximum benefit**	**Likely to be most effective TMS strategy**
Parkinson's disease	Patients with predominant motor symptoms	1. HF-rTMS to MC, less focal MC stimulation such as to leg or bilateral hand MC, and DLPFC 2. LF-rTMS to SMA
	Patients with depression	HF-rTMS on left DLPFC
Other movement disorders	Dystonia	No conclusive evidence; Low-frequency rTMS on dorsal PMC may be beneficial
	Huntington's disease	No conclusive evidence; Controversial data; SMA may be a promising target
	Tourette syndrome	LF- rTMS on SMA
	Essential tremor	LF-rTMS to cerebellum and pre-SMA
Alzheimer's Disease	Mild (including MCI) to moderate AD but not severe AD Higher education may confer advantage	1. HF-rTMS to multiple sites (Broca, right/left DLPFC, Wernicke, right/ pSAC, inferior frontal gyrus) 2. More number of sessions 3. Concurrent cognitive training
Multiple Sclerosis	RRMS with spasticity SPMS with spasticity	4. iTBS plus ET is a promising tool for motor rehabilitation of MS 5. HF-rTMS may help in improving dexterity and cognitive function 6. No recommendations yet for therapeutic use
Epilepsy	1. Patients with medically intractable epilepsy or drug-resistant epilepsy who are not surgical candidates 2. Patients ≤ 21 years 3. Patients with neocortical epilepsy or cortical dysplasia	1. LF-rTMS with figure 8 coil and targeted stimulation provides benefit 2. Routine use not recommended yet
Disorders of consciousness	MCS and UWS	1. HF-rTMS of the left M1 2. HF-rTMS or iTBS of the left DLPFC

*AD, Alzheimer's Disease; DLPFC, dorso lateral prefrontal cortex; ET, exercise therapy; HF-Rtms, High frequency transcranial magnetic stimulation; iTBS, intermittent theta burst stimulation; LF-Rtms, Low frequency TMS; MC, motor cortex; MCS, minimally conscious state; MS, multiple sclerosis; pSAC, parietal somatosensory association cortex; RRMS, relapsing/remitting MS; rTMS, repetitive TMS; SMA, supplementary motor area; SPMS, secondary progressing MS; UWS, unresponsive wakefulness syndrome*.

## Author Contributions

FS primarily did the literature review and wrote first draft. TG and AS contributed further insights and co-authored with FS the final manuscript. All authors contributed to the article and approved the submitted version.

## Conflict of Interest

AS is Chief Scientific Advisor for PlatoScience and Alphasys; as well as Director of the International Clinical TMS Certification Course (www.tmscourse.eu). The remaining authors declare that the research was conducted in the absence of any commercial or financial relationships that could be construed as a potential conflict of interest.

## Publisher's Note

All claims expressed in this article are solely those of the authors and do not necessarily represent those of their affiliated organizations, or those of the publisher, the editors and the reviewers. Any product that may be evaluated in this article, or claim that may be made by its manufacturer, is not guaranteed or endorsed by the publisher.
